# β-Mannanase Supplementation as an Eco-Friendly Feed Strategy to Reduce the Environmental Impacts of Pig and Poultry Feeding Programs

**DOI:** 10.3389/fvets.2021.732253

**Published:** 2021-10-11

**Authors:** Felipe M. W. Hickmann, Ines Andretta, Marie-Pierre Létourneau-Montminy, Aline Remus, Gabriela M. Galli, Juliano Vittori, Marcos Kipper

**Affiliations:** ^1^Departamento de Zootecnia, Universidade Federal do Rio Grande do Sul, Porto Alegre, Brazil; ^2^Département des Sciences Animales, Faculté des Sciences de l'Agriculture et de l'Alimentation, Université Laval, Québec, QC, Canada; ^3^Sherbrooke Research and Development Center, Agriculture and Agri-Food Canada, Sherbrooke, QC, Canada; ^4^Elanco Animal Health, São Paulo, Brazil

**Keywords:** swine, broiler, environment, feed, enzyme, climate change, sustainability, life-cycle assessment

## Abstract

Little is still known about the environmental impacts of exogenous enzyme supplementation in pig and poultry feeding programs. Thus, this study aimed to assess the potential environmental impacts of producing feeds for pigs and broilers by simulating the effects of β-mannanase *Hemicell*™ *HT* supplementation through energy savings during diet formulation. Life-cycle assessment standards were applied to simulate a cradle-to-feed mill gate scope. The functional units used were the production of 1 kg of the enzyme and 1 kg of feed at a feed mill gate located in Concórdia, Santa Catarina, Brazil. Climate change, eutrophication, and acidification were the chosen environmental impact categories. Energy savings through β-mannanase supplementation were assessed by different metabolizable energy (ME) matrices (45 or 90 kcal of ME/kg of feed) during diet formulation in different grain production scenarios (Southern and/or Central-West origin). A total of 28 feeds were formulated based on the nutritional requirements and feeding programs described in the Brazilian Tables for Poultry and Swine. The least-cost formulation method was used based on real price averages practiced in a local industry over 12 months. The production of 1 kg of β-mannanase was associated with the emission of 1,800 g of CO_2_-eq, 4.53 g of PO_4_-eq, and 7.89 g of SO_2_-eq. For pig feeds, β-mannanase supplementation mitigated both climate change and eutrophication impacts up to 8.5 and 1.4% (45 kcal of ME/kg of feed) or up to 16.2 and 2.7% (90 kcal of ME/kg of feed) compared to control diets formulated without the enzyme. For broiler feeds, these impacts were mitigated up to 5.6 and 1.1% (45 kcal of ME/kg of feed), respectively. On the other hand, the effect of using β-mannanase on the acidification impact was not consistent among feeds/species. Overall, β-mannanase supplementation reduced the amount of soybean oil in feed formulas, which is associated with high environmental impacts. Consequently, the potential impacts of climate change and eutrophication associated with producing feeds for pigs and broilers were substantially mitigated. These results suggest that β-mannanase supplementation is an eco-friendly feed strategy to reduce the environmental impacts of pig and poultry feeding programs.

## Introduction

Pig and poultry feeding programs require a huge amount of feed resources, with several studies indicating feeding as a major source of environmental impact (1–3). A systematic review recently developed by Andretta et al. (4) on the use of life cycle analysis confirmed the importance of feeding processes as the largest source of environmental impact associated with pig and poultry production. In their review, the relative participation of feed production in the overall greenhouse gas emissions varied from 31 to 76% or 28 to 82% for the pig and poultry databases, respectively (4). Regardless of the exact amount of impact attributed to feeding, practically all studies indicated feeding as the most important environmental impact source. These results support the hypothesis that novel feeding strategies could be used as eco-friendly strategies to mitigate the environmental impacts of pig and poultry production.

The use of exogenous enzymes has been highlighted as a promising alternative to mitigate the environmental impacts of livestock (5, 6). Nonetheless, pigs and poultry lack some enzymes, such as β-mannanase, to completely digest β-mannans commonly present in a great variety of feedstuffs, including soybean, corn DDG, sunflower, copra, and palm kernel meal-based diets. This may reduce growth performance once β-mannans are associated with increased intestinal viscosity and decreased nutrient digestibility, following an inflammatory process initiated in response to the β-mannans presence (7–9). *Hemicell*™ *HT* is a source of β-mannanase, an energy-sparing enzyme that hydrolyzes β-mannans, avoiding the inflammatory reaction (10). β-mannanase supplementation can then potentially improve the nutrient digestibility and growth performance of pigs and broilers. In addition, when an energy matrix is attributed to the enzyme during feed formulation, some resources are saved, leading to an increase in energy-use efficiency.

Despite the importance of both pig and poultry sectors in developing countries, most studies that assessed the environmental impacts of exogenous enzyme supplementation had been developed based on European and North American conditions, with limited applicability to other major pig and poultry production regions. In addition, little is still known about the environmental impacts of using β-mannanase in feeding programs. Therefore, this study aimed to assess the potential environmental impacts of producing feeds for pigs and broilers by simulating the effects of β-mannanase *Hemicell*™ *HT* supplementation through energy savings during diet formulation.

## Materials and Methods

**Figure 1:**
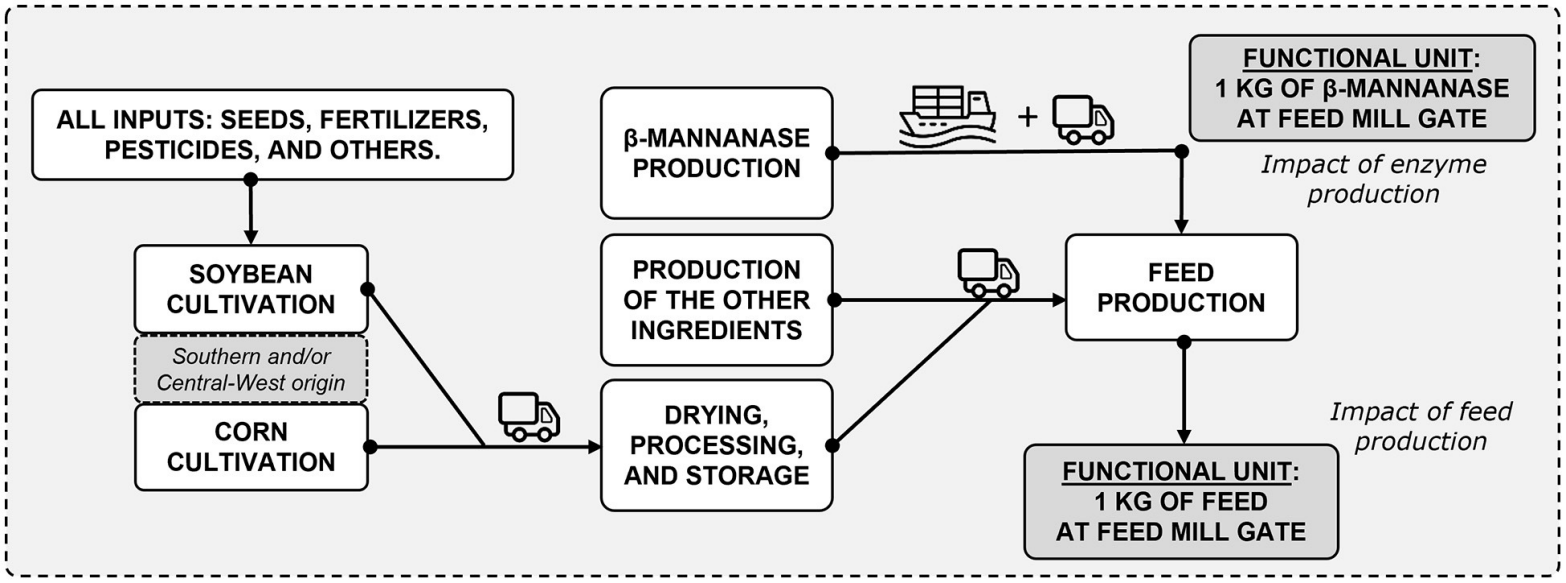
Flowchart of the pig and poultry feeding programs being assessed through life-cycle assessment standards. Crop inputs, crop production, β-mannanase production, production of the other feed ingredients, drying, processing, storage, transportation, and feed production were the main processes considered, with system boundaries including all sub-processes.

Environmental impacts were assessed according to life-cycle assessment (LCA) standards based on four interrelated steps, described by Guinée (11) as (i) goal and scope definition, (ii) life cycle inventory, (iii) life cycle impact assessment, and (iv) interpretation of results. Brazil was chosen because it is a large producer and exporter of pork and chicken meat. For this study, in a cradle-to-feed mill gate scope, the major stages considered in the model were the production of β-mannanase *Hemicell*™ *HT*, the production of feed ingredients from plant sources (corn and soybean meal), and the production of the other feed ingredients (including amino acids, limestone, dicalcium phosphate, salt, and vitamin-mineral premix). Drying and processing in the feed industry as well as transportation were also considered, as illustrated in Figure 1. The functional unit used to study the environmental cost of feedstuff (especially for enzyme or grain production) was 1 kg of each ingredient at the feed factory. The functional unit used to study potential environmental impacts associated with feeds was 1 kg of feed manufactured and ready to be delivered to the farm (at the feed mill gate). The animal phase was not included in the scope due to limitations in data availability, mainly on the impact of β-mannanase supplementation (e.g., enteric fermentation). In addition, previous evidence showed no differences in performance (i.e., feed efficiency and nutrient metabolism) when supplementing β-mannanase with an energy matrix attributed during diet formulation (12).

### Description of the Pig and Poultry Production Systems Evaluated

An inventory for β-mannanase *Hemicell*™ *HT* production was developed using detailed information provided by the manufacturer company (Elanco Animal Health, Greenfield, IN, US). Energy requirements (electricity, heating, and cooling) and emissions of CO_2_ during enzyme production were adapted from Gilpin et al. (13). Simulations considered enzyme production in the industrial plant at Greenfield (Delphos, US), followed by road transportation using trucks and marine transportation using cargo ships until arriving at the feed factory in Brazil.

All other simulations were developed considering a feed mill located in Concórdia (Santa Catarina, Brazil) since it represents a traditional pig and poultry producing region in Southern Brazil. Grain production was independently characterized in both Central-West (CW) and Southern (SO) regions in Brazil, as described by Andretta et al. (14). Crop farm locations were chosen based on rankings of the largest corn- and soybean-producing municipalities within each region (15). Agricultural practices for grain production and the models used to calculate their emissions were adapted from Alvarenga (16), Alvarenga et al. (17), and Prudêncio da Silva et al. (18). The land transformation was estimated based on the data provided by Alvarenga (16), following the methodology described by Prudêncio da Silva et al. (18). Grain yield data were obtained from the Brazilian Institute of Geography and Statistics (15) for each municipality.

As pointed out by Prudêncio da Silva et al. (18), the environmental footprint of grain production depends on the Brazilian region being considered for crop cultivation. Thus, three geographic scenarios were simulated based on different crop cultivation locations: CW-CW, in which only grains from CW were used to produce feeds; CW-SO, in which soybean from CW and corn from SO were used to produce feeds; and SO-SO, in which only grains from SO were used to produce feeds. These scenarios differed in terms of road transportation distances, agricultural practices, and deforestation impact on recently opened agricultural frontiers (deforestation was assumed for the CW region but not included for the characterization of production in the SO region). Information from the Ecoinvent database (v. 3.0, Swiss Center for Life Cycle Inventories, Dübendorf, Switzerland) was used to characterize soybean oil production. A process with solvent was applied for obtaining the product, with no geographical scenarios considered for oil production.

The impact of phytase supplementation was simulated considering the information provided by Nielsen et al. (5). The scope of synthetic amino acid production was adapted from Mosnier et al. (19), distinguishing amino acids produced by chemical synthesis (DL-methionine) from those produced by fermentation (L-lysine, L-threonine, L-tryptophane, and L-valine). All other feed ingredients were based on available databases. The Ecoinvent database (v. 3.0, Swiss Center for Life Cycle Inventories, Dübendorf, Switzerland) was used to characterize the production of meat and bone meal, sodium chloride, and limestone. The environmental impacts of vitamin-mineral trace elements were assumed to be equal to those of limestone. On the other hand, the environmental impacts of soybean protein isolate and whey were based on the AgriFootPrint database (v. 5.0, Blonk Consultants, Gouda, The Netherlands).

Grain processing and storage conditions were adapted from previous reports (20, 21). Transportation of grains (from the farm to the feed factory), other ingredients (from the industry to the feed factory), and feeds (from the feed factory to the pig farm) were assumed to have been done by truck, with the exception of enzymes that also included marine transportation. The Google Earth software (Google Inc., Mountain View, CA) was used to estimate transportation distances. Information from the Agri-footprint database (v. 5, Blonk Consultants, Gouda, The Netherlands) was used to simulate the impact of transportation.

### Feeding Practices

Ingredients commonly used in Brazil were used to formulate feeds. Soybean meal was the major protein source, combined with corn and refined soybean oil as the major energy suppliers. A total of 28 feeds (Tables 1–4) were formulated based on the nutritional requirements and feeding programs described in the Brazilian Tables for Poultry and Swine (22). For pigs, pre-starter, starter, growing I, growing II, finishing I, and finishing II feeds were formulated based on animals with 33–42, 49–63, 70–84, 91–105, 112–133, and 140–161 days of age, respectively, and 10.8, 22.5, 40, 60, 85, and 112.5 kg of body weight, respectively. For broilers, starter I, starter II, growing I, growing II, and finishing feeds were formulated based on animals with 1–7, 8–21, 22–33, 34–42, and 43–46 days of age, respectively, and 0.14, 0.59, 1.65, 2.78, and 3.48 kg of body weight, respectively. These feeds were formulated considering animals for slaughter only (excluding breeding animals) once they represent most of the feed produced in pig and poultry feeding programs, with complex and simple formulas being simulated for nursery pigs.

**Table 1:** Composition of nursery pig feeds^a^.

	**Pre-starter**				**Starter**	
	**Complex**		**Simple**		
	**Control**	**β-mannanase^b^**	**Control**	**β-mannanase**	**Control**	**β-mannanase**
**Ingredient (as-fed basis), %**				
Corn	57.08	58.25	54.30	55.47	55.08	56.25
Soybean meal	12.00	11.88	25.00	24.88	37.87	37.75
Soybean oil	1.61	0.52	2.52	1.44	3.35	2.27
Meat and bone meal	5.00	5.00	5.00	5.00	-	-
Soybean isolate protein	8.13	8.13	4.30	4.30	-	-
Spray-dried plasma	5.00	5.00	2.50	2.50	-	-
Whey	10.00	10.00	5.00	5.00	-	-
L-lysine HCL	0.11	0.11	0.17	0.17	0.29	0.29
DL-methionine	0.10	0.10	0.11	0.11	0.13	0.13
L-threonine	0.05	0.05	0.09	0.09	0.12	0.12
L-valine	0.02	0.02	0.02	0.02	0.05	0.05
Salt	0.22	0.22	0.21	0.21	0.19	0.19
Limestone	0.41	0.41	0.42	0.42	1.05	1.05
Dicalcium phosphate	-	-	-	-	1.13	1.13
Vitamin-mineral premix	0.50	0.50	0.50	0.50	0.50	0.50
β-mannanase	-	0.03	-	0.03	-	0.03
**Calculated chemical composition** ^c^				
Crude protein, %	23.81	23.81	24.08	24.08	21.88	21.88
SID^d^ lysine, %	1.35	1.35	1.35	1.35	1.28	1.28
Metabolizable energy, kcal/kg	3,375	3,375	3,375	3,375	3,350	3,350
Digestible phosphorus, %	0.53	0.53	0.51	0.51	0.45	0.45

a * Pre-starter and starter feeds were formulated based on animals with 33–42 and 49–63 days of age, respectively, with 10.8 and 22.5 kg of body weight on average, respectively*.

b * 90 kcal of metabolizable energy/kg of feed was the energy matrix attributed to the enzyme during diet formulation of nursery piglet feeds*.

c * Values were estimated considering the Brazilian Tables for Poultry and Swine (22)*.

d * Standardized ileal digestible*.

**Table 2:** Composition of growing pig feeds^a^.

	**Growing I**			**Growing II**		
	**Control**	***β*** **-mannanase^b^**		**Control**	***β*** **-mannanase**	
		**45 kcal**	**90 kcal**		**45 kcal**	**90 kcal**
**Ingredient (as-fed basis), %**				
Corn	68.60	69.59	70.63	73.69	74.67	75.72
Soybean meal	23.43	23.31	23.19	19.73	19.61	19.49
Soybean oil	2.22	1.32	0.41	1.89	1.00	0.08
Meat and bone meal	3.35	3.34	3.33	2.39	2.38	2.37
L-lysine HCL	0.49	0.49	0.49	0.46	0.46	0.46
DL-methionine	0.19	0.19	0.19	0.15	0.15	0.15
L-threonine	0.19	0.19	0.19	0.17	0.17	0.16
L-tryptophane	0.06	0.06	0.06	0.05	0.05	0.05
L-valine	0.07	0.07	0.07	0.06	0.05	0.05
Salt	0.39	0.39	0.39	0.38	0.38	0.38
Limestone	0.51	0.52	0.52	0.54	0.54	0.55
Vitamin-mineral premix	0.50	0.50	0.50	0.50	0.50	0.50
Phytase	0.01	0.01	0.01	0.01	0.01	0.01
β-mannanase	-	0.03	0.03	-	0.03	0.03
**Calculated chemical composition^c^**				
Crude protein, %	18.31	18.33	18.35	16.51	16.53	16.56
SID^d^ lysine, %	1.16	1.16	1.16	1.03	1.03	1.03
Metabolizable energy, kcal/kg	3,350	3,350	3,350	3,350	3,350	3,350
Digestible phosphorus, %	0.38	0.38	0.38	0.33	0.33	0.33

a * Growing I and growing II feeds were formulated based on animals 70–84 and 91–105 days of age, respectively, with 40 and 60 kg of body weight on average, respectively*.

b * 45 and 90 kcal of metabolizable energy/kg of feed were the energy matrices attributed to the enzyme during diet formulation of growing pig feeds*.

c * Values were estimated considering the Brazilian Tables for Poultry and Swine (22)*.

d * Standardized ileal digestible*.

**Table 3:** Composition of finishing pig feeds^a^.

	**Finishing I**			**Finishing II**		
	**Control**	***β*** **-mannanase^b^**		**Control**	***β*** **-mannanase**	
		**45 kcal**	**90 kcal**		**45 kcal**	**90 kcal**
**Ingredient (as-fed basis), %**				
Corn	78.98	79.97	78.66	84.68	85.67	84.61
Soybean meal	15.56	15.45	17.51	10.76	10.64	11.58
Soybean oil	1.60	0.71	-	1.33	0.43	-
Meat and bone meal	1.65	1.64	1.58	1.17	1.16	1.14
L-lysine HCL	0.43	0.43	0.37	0.39	0.39	0.36
DL-methionine	0.12	0.12	0.10	0.07	0.07	0.06
L-threonine	0.14	0.14	0.11	0.10	0.10	0.09
L-tryptophane	0.05	0.05	0.04	0.04	0.04	0.04
L-valine	0.04	0.04	0.00	0.02	0.02	0.00
Salt	0.36	0.36	0.36	0.35	0.35	0.35
Limestone	0.57	0.57	0.74	0.59	0.59	1.23
Vitamin-mineral premix	0.50	0.50	0.50	0.50	0.50	0.50
Phytase	0.01	0.01	0.01	0.01	0.01	0.01
β-mannanase	-	0.03	0.03	-	0.03	0.03
**Calculated chemical composition^c^**				
Crude protein, %	14.62	14.65	15.34	12.58	12.60	12.88
SID^d^ lysine, %	0.90	0.90	0.90	0.75	0.75	0.75
Metabolizable energy, kcal/kg	3,350	3,350	3,350	3,350	3,350	3,350
Digestible phosphorus, %	0.28	0.28	0.28	0.25	0.25	0.25

a * Finishing I and finishing II feeds were formulated based on animals with 112–133 and 140–161 days of age, respectively, with 85 and 112.5 kg of body weight on average, respectively*.

b * 45 and 90 kcal of metabolizable energy/kg of feed were the energy matrices attributed to the enzyme during diet formulation of finishing pig feeds*.

c * Values were estimated considering the Brazilian Tables for Poultry and Swine (22)*.

d * Standardized ileal digestible*.

**Table 4:** Composition of broiler feeds^a^.

	**Starter**				**Growing**				**Finishing**	
	**I**		**II**		**I**		**II**			
	**Control**	**βM^b^**	**Control**	**βM**	**Control**	**βM**	**Control**	**βM**	**Control**	**βM**
**Ingredient (as-fed basis), %**								
Corn	43.59	44.54	45.38	46.32	50.52	51.44	59.20	60.12	63.80	64.73
Soybean meal	46.11	45.96	43.60	43.46	38.02	37.90	30.87	30.76	26.69	26.57
Soybean oil	5.50	4.66	6.60	5.76	7.13	6.30	6.24	5.40	6.13	5.30
L-lysine HCL	0.22	0.22	0.22	0.23	0.35	0.35	0.34	0.34	0.34	0.34
DL-methionine	0.41	0.41	0.39	0.39	0.38	0.38	0.31	0.30	0.26	0.26
L-threonine	0.07	0.07	0.06	0.06	0.09	0.09	0.06	0.06	0.05	0.04
L-valine	0.02	0.02	0.02	0.02	0.05	0.05	0.04	0.04	0.03	0.03
Salt	0.22	0.22	0.21	0.21	0.19	0.19	0.17	0.17	0.16	0.16
Limestone	1.07	1.07	0.96	0.97	0.91	0.92	0.76	0.76	0.69	0.69
Dicalcium phosphate	1.84	1.84	1.61	1.61	1.41	1.41	1.07	1.07	0.92	0.91
Sodium bicarbonate	0.45	0.45	0.45	0.45	0.44	0.44	0.44	0.44	0.44	0.44
Vitamin-mineral premix	0.50	0.50	0.50	0.50	0.50	0.50	0.50	0.50	0.50	0.50
β-mannanase	-	0.03	-	0.03	-	0.03	-	0.03	-	0.03
**Calculated chemical composition** ^c^								
Crude protein, %	24.56	24.57	23.56	23.57	21.55	21.57	18.95	18.97	17.39	17.41
SID^d^ lysine, %	1.36	1.36	1.31	1.31	1.24	1.24	1.07	1.07	0.97	0.97
Metabolizable energy, kcal/kg	3,000	3,000	3,100	3,100	3,200	3,200	3,250	3,250	3,300	3,300
Digestible phosphorus, %	0.48	0.48	0.43	0.43	0.38	0.38	0.31	0.31	0.27	0.27

a * Starter I, starter II, growing I, growing II, and finishing feeds were formulated based on animals with 1–7, 8–21, 22–33, 34–42, and 43–46 days of age, respectively, and 0.14, 0.59, 1.65, 2.78, and 3.48 kg of body weight, respectively*.

b * β-mannanase supplementation, 45 kcal of metabolizable energy/kg of feed was the energy matrix attributed to the enzyme during diet formulation of broiler feeds*.

c * Values were estimated considering the Brazilian Tables for Poultry and Swine (22)*.

d * Standardized ileal digestible*.

During diet formulation, the replacement of soybean oil was performed automatically by the formulation software (Formula 2000, Optimal Informatica, Campinas, Brazil). The least-cost formulation method was used considering real price averages practiced in a local industry over 12 months. The nutritional composition of the ingredients was obtained from the Brazilian Tables for Poultry and Swine (22). The metabolizable energy (ME) matrix of β-mannanase was chosen based on the most common values applied to the Brazilian industry (45 or 90 kcal of ME/kg of feed, depending on the species and rearing phase). Both matrices were simulated for growing-finishing pigs. While 45 kcal of ME/kg of feed was the energy matrix simulated for broilers, 90 kcal of ME/kg of feed was the energy matrix simulated for nursery pigs.

### Modeling Environmental Impacts

Inputs and outputs were defined for each step of the life cycle and organized in a model using the SimaPro software (v. 9.1.1.1, PRE-Consultants, Amersfoort, The Netherlands). Environmental impacts related to capital assets (machinery, equipment, and buildings) were not considered in the model. The allocation of environmental burdens to by-products was based on economic criteria. The functional units considered were 1 kg of the enzyme at the feed mill gate to account for the environmental impacts associated with β-mannanase supplementation and 1 kg of feed at the feed mill gate to evaluate the impact of feed production and the grain production scenarios.

Climate change, eutrophication, and acidification were the chosen environmental impact categories, the most common impact categories used to assess the environmental impacts of pig and poultry production (4). Results were obtained for each environmental impact category, stating the resources used in each production system and the aggregate emissions of each substance with the respective characterization factor. The CML-IA baseline method was used through the SimaPro software to calculate the environmental impacts (CO_2_-eq, PO_4_-eq, and SO_2_-eq). Changes in potential environmental impacts associated with β-mannanase supplementation were estimated considering the total amount of feed used to raise a pig or a broiler (from hatch/weaning until slaughter, excluding feeds for breeders). For this simulation, feed intake was estimated using the Brazilian Tables for Poultry and Swine (22).

### Simulating Another Formulation Strategy

Data obtained from a previous study (12) was also simulated to consider a different energy matrix released through β-mannanase supplementation. Even though formulas had the same ingredient base (corn and soybean meal), the formulation procedure differs from the one described in this study. Lv et al. (12) did not use the least-cost formulation method to formulate diets, with 150 kcal of digestible energy (DE)/kg of feed being released in β-mannanase supplemented diets.

### Simulating the Environmental Impacts Associated With Energy Reduction

Under Brazilian pig and poultry production conditions, the main change in ingredients after the inclusion of β-mannanase during diet formulation is the reduction of soybean oil content. The association between the reduction of soybean oil content in feed formulas and the estimated mitigation of environmental impacts was evaluated using regression analysis. The significance (*P* < 0.05) of each equation term was evaluated before interpretation. Since species was not significant, one regression was created for both pigs and broilers. Analyses were performed using the Minitab 20.2.0 software (23).

Another simulation was performed to estimate the minimum amount of energy matrix necessary to mitigate the environmental cost of producing and transporting the enzyme. In this case, due to changes in diet formulation with β-mannanase supplementation, the soybean oil impact was fully replaced by the impact of corn in the simulation. ME values for soybean oil and corn were those proposed in the Brazilian Tables for Poultry and Swine (22). Information from the Ecoinvent database (v. 3.0, Swiss Center for Life Cycle Inventories, Dübendorf, Switzerland) was used to characterize the soybean oil production. The environmental impact of oil reduction was estimated considering other references to include variability in the simulations (AgriFootPrint v. 5.0, Blonk Consultants, Gouda, The Netherlands), all based on Brazilian production scenarios.

## Results

The production of corn (functional unit: 1 kg at the feed mill gate) in the Southern region led to the emission of 491 g of CO_2_-eq, 3.78 g of PO_4_-eq, and 9.98 g of SO_2_-eq. For the Central-West region, corn showed a higher impact concerning climate change (601 g of CO_2_-eq; +22%) and eutrophication (3.88 g of PO_4_-eq; +3%) but a lower impact concerning acidification (8.92 g of PO_4_-eq;−11%) compared to the Southern region. The production of soybean meal (1 kg at the feed mill gate) in the Southern region was associated with the emission of 533 g of CO_2_-eq, 5.82 g of PO_4_-eq, and 2.62 g of SO_2_-eq. In comparison with the Southern region, soybean meal from the Central-West region showed a higher impact concerning climate change (1,110 g of CO_2_-eq; +108%) and acidification (5.43 g of SO_2_-eq; +107%) but a lower impact concerning eutrophication (5.64 g of PO_4_-eq;−3%). These differences among ingredient origins are highlighted in the impacts of producing complete feed formulas (functional unit: 1 kg at the feed mill gate) for pigs and broilers, which are presented in Tables 5–7.

**Table 5:** Potential environmental impacts of control feeds^a^ (1 kg at feed mill gate, formulated without β-mannanase) for nursery piglets in different grain production scenarios.

	**Pre-Starter**		**Starter**
	**Complex**	**Simple**	
**SO-SO scenario** ^b^			
Climate change, g CO_2_-eq	1,266	994	695
Eutrophication, g PO_4_-eq	4.08	4.38	4.66
Acidification, g SO_2_-eq	7.79	7.14	6.78
**CW-SO scenario** ^c^			
Climate change, g CO_2_-eq	1,336	1,138	914
Eutrophication, g PO_4_-eq	4.06	4.34	4.59
Acidification, g SO_2_-eq	8.13	7.84	7.85
**CW-CW scenario** ^d^			
Climate change, g CO_2_-eq	1,398	1,198	974
Eutrophication, g PO_4_-eq	4.12	4.39	4.65
Acidification, g SO_2_-eq	7.53	7.27	7.26

a *Pre-starter and starter feeds were formulated based on animals with 33–42 and 49–63 days of age, respectively, with 10.8 and 22.5 kg of body weight on average, respectively*.

b *SO-SO scenario: Soybean and corn produced in Southern Brazil*.

c *CW-SO scenario: Soybean produced in Central-West Brazil and corn produced in Southern Brazil*.

d *CW-CW scenario: Soybean and corn produced in Central-West Brazil*.

**Table 6:** Potential environmental impacts of control feeds^a^ (1 kg at feed mill gate, formulated without β-mannanase) for growing-finishing pigs in different grain production scenarios.

	**Growing**		**Finishing**	
	**I**	**II**	**I**	**II**
**SO-SO scenario** ^b^				
Climate change, g CO_2_-eq	633	614	596	577
Eutrophication, g PO_4_-eq	4.33	4.25	4.15	4.03
Acidification, g SO_2_-eq	7.81	8.18	8.55	8.95
**CW-SO scenario** ^c^				
Climate change, g CO_2_-eq	768	728	686	639
Eutrophication, g PO_4_-eq	4.29	4.21	4.12	4.01
Acidification, g SO_2_-eq	8.47	8.73	8.99	9.25
**CW-CW scenario** ^d^				
Climate change, g CO_2_-eq	844	809	773	732
Eutrophication, g PO_4_-eq	4.36	4.28	4.20	4.10
Acidification, g SO_2_-eq	7.74	7.95	8.15	8.36

a *Growing I, growing II, finishing I, and finishing II feeds were formulated based on animals 70–84, 91–105, 112–133, and 140–161 days of age, respectively, with 40, 60, 85, and 112.5 kg of body weight on average, respectively*.

b *SO-SO scenario: Soybean and corn produced in Southern Brazil*.

c *CW-SO scenario: Soybean produced in Central-West Brazil and corn produced in Southern Brazil*.

d *CW-CW scenario: Soybean and corn produced in Central-West Brazil*.

**Table 7:** Potential environmental impacts of control feeds^a^ (1 kg at feed mill gate, formulated without β-mannanase) for broilers in different grain production scenarios.

	**Starter**		**Growing**		**Finishing**
	**I**	**II**	**I**	**II**	
**SO-SO scenario** ^b^					
Climate change, g CO_2_-eq	816	877	910	857	848
Eutrophication, g PO_4_-eq	4.94	4.97	4.90	4.71	4.62
Acidification, g SO_2_-eq	5.96	6.13	6.56	7.17	7.50
**CW-SO scenario** ^c^					
Climate change, g CO_2_-eq	1,082	1,128	1,130	1,035	1,002
Eutrophication, g PO_4_-eq	4.86	4.89	4.83	4.66	4.58
Acidification, g SO_2_-eq	7.26	7.35	7.63	8.04	8.25
**CW-CW scenario** ^d^					
Climate change, g CO_2_-eq	1,130	1,178	1,185	1,100	1,072
Eutrophication, g PO_4_-eq	4.90	4.94	4.88	4.72	4.64
Acidification, g SO_2_-eq	6.80	6.87	7.09	7.41	7.58

a *Starter I, starter II, growing I, growing II, and finishing feeds were formulated based on animals with 1–7, 8–21, 22–33, 34–42, and 43–46 days of age, respectively, and 0.14, 0.59, 1.65, 2.78, and 3.48 kg of body weight, respectively*.

b *SO-SO scenario: Soybean and corn produced in Southern Brazil*.

c *CW-SO scenario: Soybean produced in Central-West Brazil and corn produced in Southern Brazil*.

d *CW-CW scenario: Soybean and corn produced in Central-West Brazil*.

**Figure 2:**
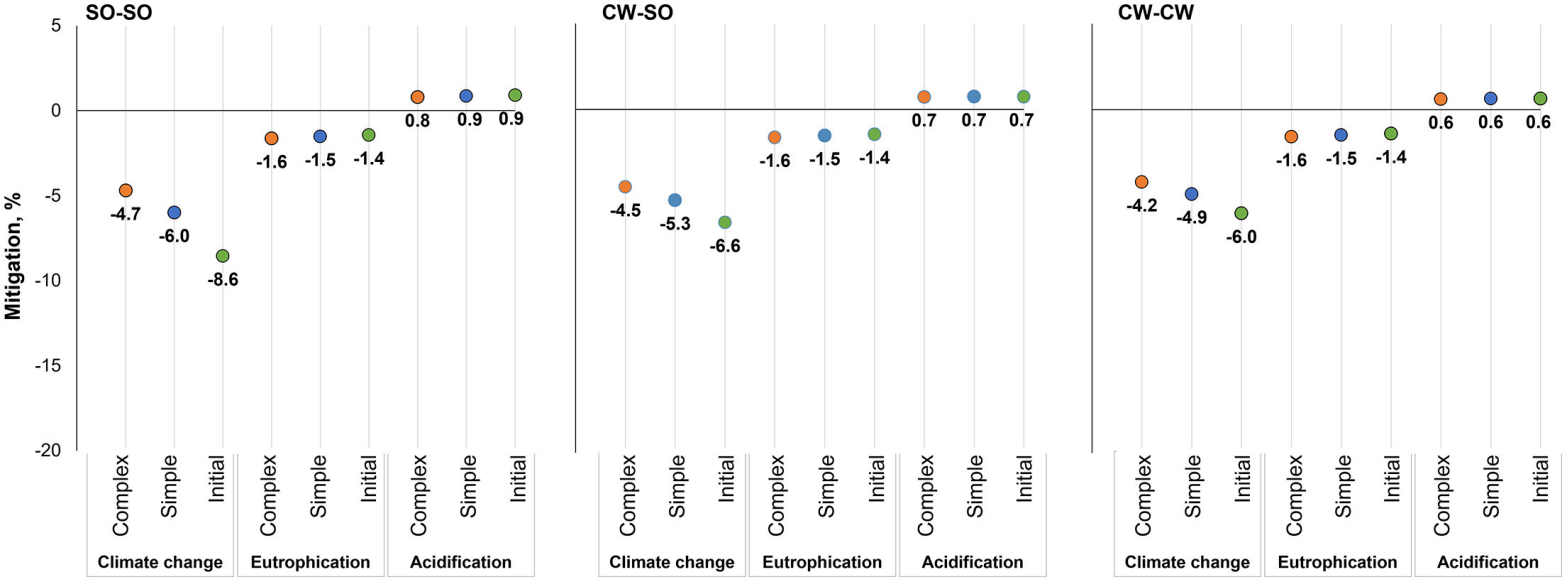
Potential environmental impact mitigation associated with the production of piglet feeds, considering energy savings from an energy matrix of 90 kcal of ME/kg of feed released by β-mannanase supplementation in different grain production scenarios; SO-SO, Soybean and corn produced in Southern Brazil; CW-SO, Soybean produced in Central-West Brazil and corn produced in Southern Brazil; CW-CW, Soybean and corn produced in Central-West Brazil.

**Figure 3:**
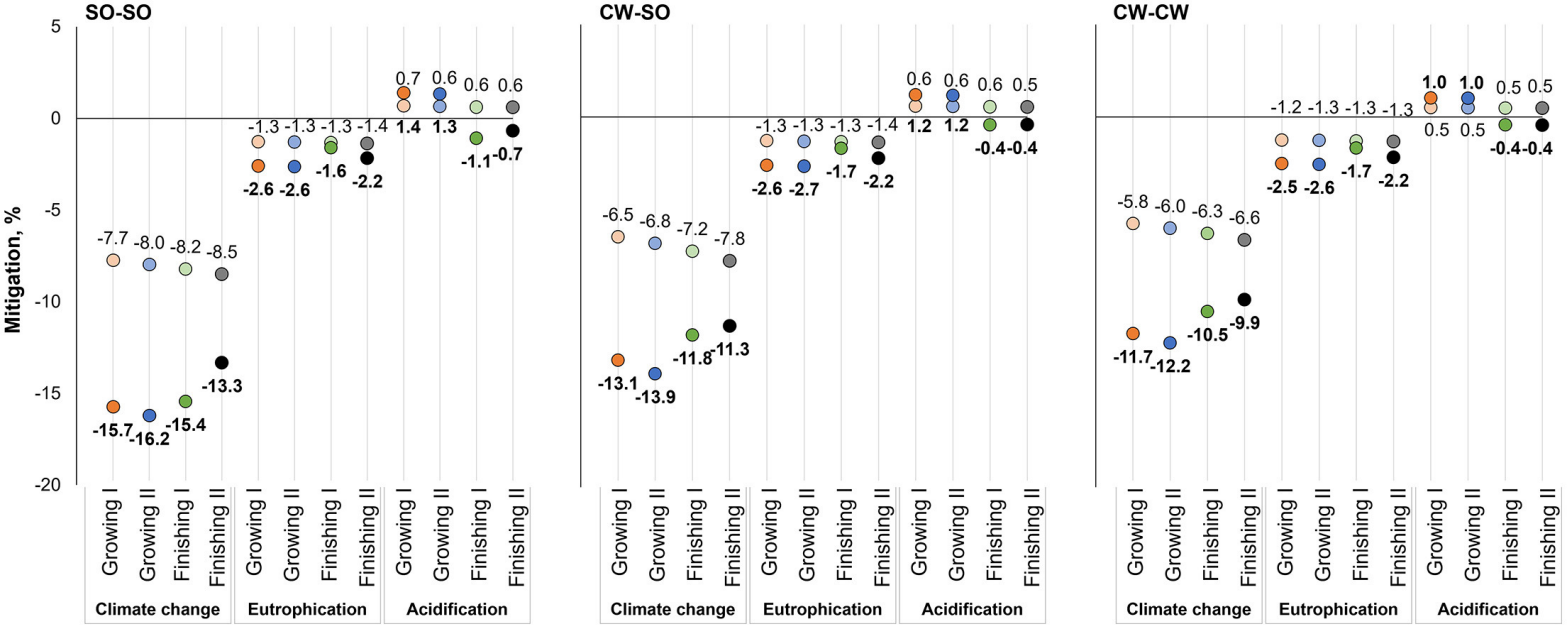
Potential environmental impact mitigation associated with the production of growing-finishing pig feeds, considering energy savings from two energy matrices (45 kcal of ME/kg of feed, light circles; 90 kcal of ME/kg of feed, dark circles) released by β-mannanase supplementation in different grain production scenarios; SO-SO, Soybean and corn produced in Southern Brazil; CW-SO, Soybean produced in Central-West Brazil and corn produced in Southern Brazil; CW-CW, Soybean and corn produced in Central-West Brazil.

**Figure 4:**
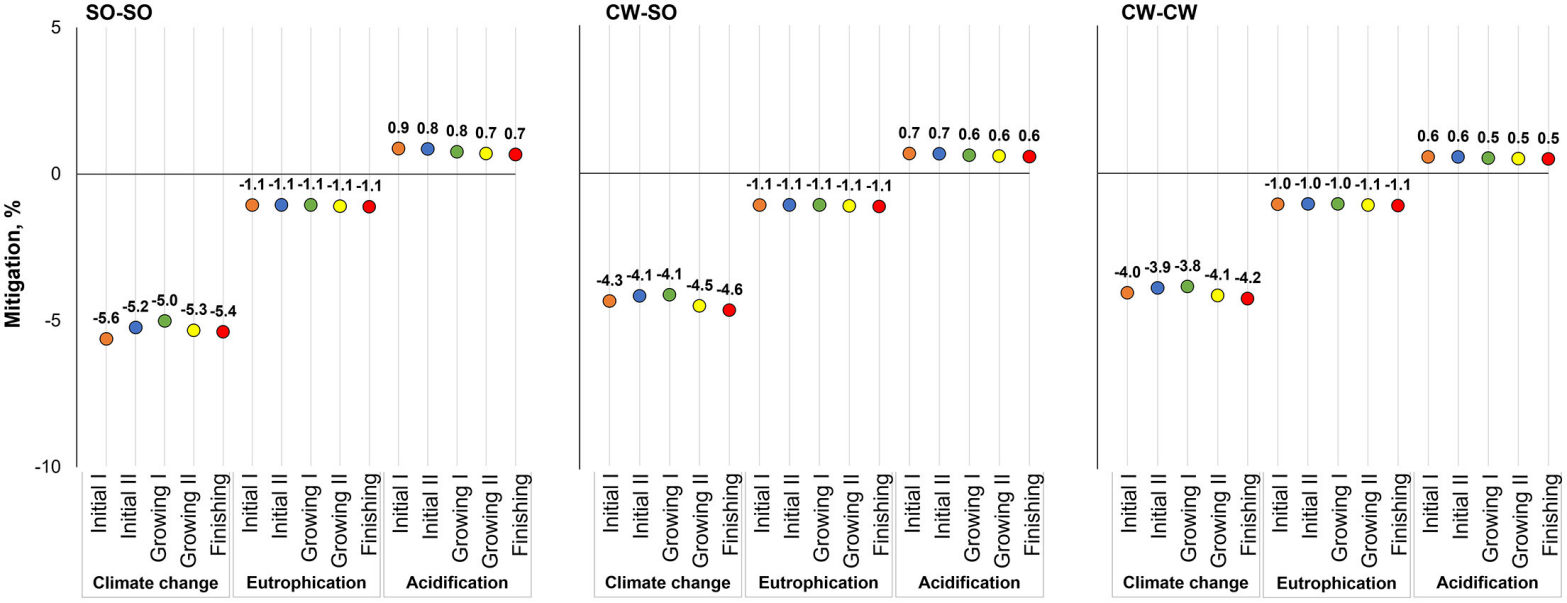
Potential environmental impact mitigation associated with the production of broiler feeds, considering energy savings from an energy matrix of 45 kcal of ME/kg of feed released by β-mannanase supplementation in different grain production scenarios; SO-SO, Soybean and corn produced in Southern Brazil; CW-SO, Soybean produced in Central-West Brazil and corn produced in Southern Brazil; CW-CW, Soybean and corn produced in Central-West Brazil.

The production of β-mannanase (1 kg at the feed mill gate) was associated with the emission of 1,800 g of CO_2_-eq, 4.53 g of PO_4_-eq, and 7.89 g of SO_2_-eq. When feeds were reformulated considering the inclusion of the enzyme and its energy matrix, there were some modifications in the ingredient use, which lead to changes in the potential environmental impact associated with the same functional unit of feed production (Figures 2–4). The use of β-mannanase associated with an energy matrix of 90 kcal of ME/kg for pre-starter diets leads to the mitigation of 59 g of CO_2_-eq per kg of produced feed, representing a greater percentage reduction in simple than complex diets. Using β-mannanase in growing-finishing pig feeds reduced the potential impact of climate change up to 16.2% when using an energy matrix of 90 kcal of ME/kg and up to 8.5% when considering 45 kcal of ME/kg. The eutrophication impact of producing the same feeds was reduced up to 2.7% when using an energy matrix of 90 kcal of ME/kg, while the reduction reached up to 1.4% when using an energy matrix of 45 kcal of ME/kg. The climate change impact of producing feeds for broilers was reduced up to 5.6%, while the eutrophication impact was reduced by up to 1.1%.

The effect of using β-mannanase on the acidification impact was not consistent among feeds. Although mitigation on the potential acidification impact was observed in feeds for finishing pigs, the use of β-mannanase (and the consequent modifications in other ingredients inclusion) increased the acidification impact associated with most other pig feeds (even when formulating considering an energy matrix of 90 kcal of ME/kg) and with all broiler feeds evaluated.

**Figure 5:**
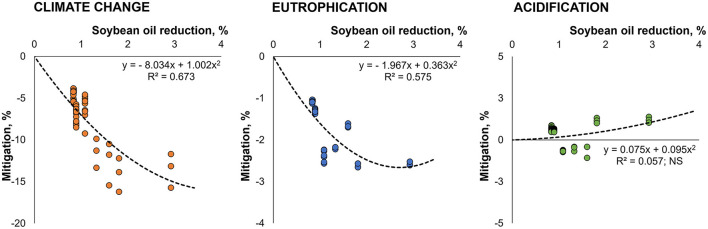
Potential environmental impact mitigation associated with the production of pig and broiler feeds due to soybean oil reduction through energy savings released by β-mannanase supplementation. For this regression analysis, species was not significant (*P* > 0.05) in the model. In addition, for the acidification impact, all terms were not significant in the regression equation.

The use of β-mannanase allowed a reduction in the amount of soybean oil in the feed formulas, which is associated with a high environmental impact. Consequently, the potential impacts of climate change and eutrophication were mitigated. A quadratic regression explained the association between soybean oil reduction in feed formulas (as a consequence of β-mannanase supplementation) and the mitigation of both climate change and eutrophication impacts (Figure 5).

The mitigation effect of β-mannanase supplementation is comparable between species when the total amount of feed used to raise a pig or a broiler is considered in the simulation (i.e., the feeding program, considering the feed used from hatch/weaning until slaughter, excluding breeding phases; Table 8). β-mannanase supplementation produced greater changes on the potential impact of climate change (reduction of up to 4.7% in feeding programs for pigs and 5.2% in feeding programs for broilers) compared to the eutrophication (−1.6% in pigs and −1.1% in broiler). Changes in the acidification impact associated with β-mannanase supplementation are positive (i.e., increased environmental impact), however, lower than 1% in all studied scenarios. When simulating the impacts using another formulation strategy (Table 9), mitigation was higher: the potential impact of climate change was reduced up to 18%, while eutrophication and acidification were mitigated by 6 and 4%, respectively. However, it is hard to compare results since they originally used different ingredients and formulation methods.

**Table 8:** Changes in the environmental impacts associated with β-mannanase supplementation when the total amount of feed used to raise a pig or a broiler (from hatch/weaning until slaughter, excluding breeding phases) is considered in the simulation^a^.

	**Pig**	**Broiler**
**SO-SO scenario** ^b^		
Climate change, kg CO_2_-eq	−11.14 (−4.7%)	−0.271 (−5.2%)
Eutrophication, g PO_4_-eq	−12.56 (−1.6%)	−0.308 (−1.1%)
Acidification, g SO_2_-eq	+11.95 (+0.8%)	+0.297 (+0.7%)
**CW-SO scenario** ^c^		
Climate change, kg CO_2_-eq	−11.29 (−4.5%)	−0.276 (−4.3%)
Eutrophication, g PO_4_-eq	−12.51 (−1.6%)	−0.307 (−1.1%)
Acidification, g SO_2_-eq	+11.23 (+0.7%)	+0.277 (+0.6%)
**CW-CW scenario** ^d^		
Climate change, kg CO_2_-eq	−11.04 (−4.2%)	−0.270 (−4.0%)
Eutrophication, g PO_4_-eq	−12.29 (−1.6%)	−0.301 (−1.1%)
Acidification, g SO_2_-eq	+8.86 (+0.6%)	+0.219 (+0.5%)

a *Feed intake for each animal phase was estimated using the Brazilian Tables for Poultry and Swine (22). Values indicate the total amount mitigated/increased when β-mannanase is used in the formulations, followed by the percentage change compared to scenarios without β-mannanase supplementation*.

b *SO-SO scenario: Soybean and corn produced in Southern Brazil*.

c *CW-SO scenario: Soybean produced in Central-West Brazil and corn produced in Southern Brazil*.

d *CW-CW scenario: Soybean and corn produced in Central-West Brazil*.

**Table 9:** Potential environmental impacts of feeds (1 kg at feed mill gate) formulated for growing pigs based on the 150 kcal digestible energy reduction per kg of feed, with β-mannanase supplemented diets compared to control diets.

	**Treatments^a^**		**Mitigation, %**
	**Control**	**β-mannanase**	
**SO-SO scenario** ^b^			
Climate change, g CO_2_-eq	587.19	479.41	−18
Eutrophication, g PO_4_-eq	4.27	3.99	−6
Acidification, g SO_2_-eq	7.88	7.56	−4
**CW-SO scenario** ^c^			
Climate change, g CO_2_-eq	717.01	596.31	−17
Eutrophication, g PO_4_-eq	4.23	3.96	−6
Acidification, g SO_2_-eq	8.42	8.13	−4
**CW-CW scenario** ^d^			
Climate change, g CO_2_-eq	790.11	666.82	−16
Eutrophication, g PO_4_-eq	4.29	4.02	−6
Acidification, g SO_2_-eq	7.72	7.45	−4

a *Control formula contains 3,400 kcal of digestible energy and formula supplemented with β-mannanase, considering an energy matrix of 150 kcal of digestible energy/kg of feed. Treatments proved to have similar performance and digestibility (12)*.

b *SO-SO scenario: Soybean and corn produced in Southern Brazil*.

c *CW-SO scenario: Soybean produced in Central-West Brazil and corn produced in Southern Brazil*.

d *CW-CW scenario: Soybean and corn produced in Central-West Brazil*.

**Figure 6:**
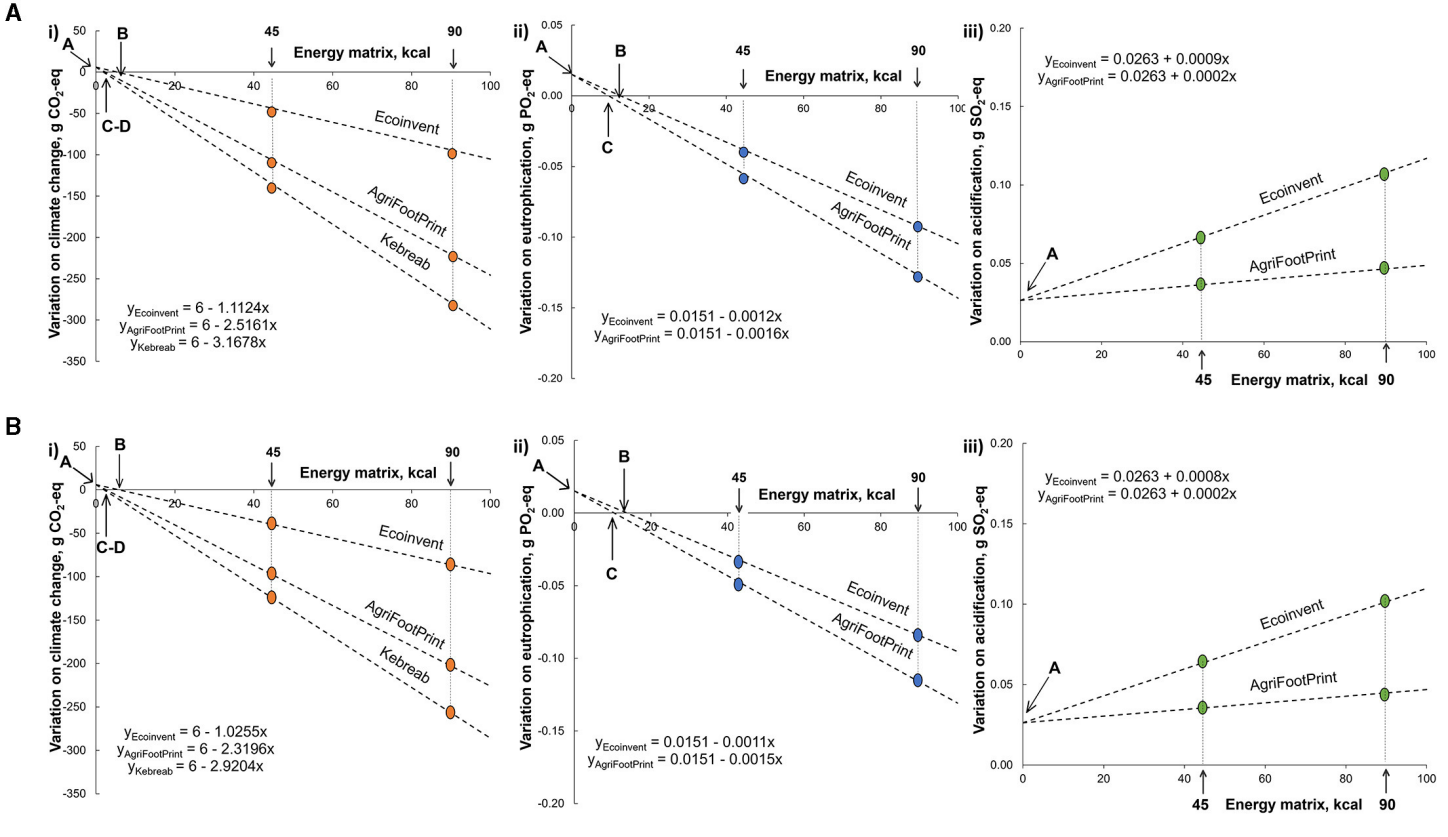
Minimum energy matrix necessary to mitigate the environmental cost associated with β-mannanase supplementation in pig **(A)** and broiler **(B)** feeds. Within each panel, the letter A indicates the environmental cost of β-mannanase supplementation for 1 kg of feed, while letters B (Ecoinvent database v. 3.0, Swiss Center for Life Cycle Inventories, Dübendorf, Switzerland), C (AgriFootPrint v. 5.0, Blonk Consultants, Gouda, The Netherlands), and D [Kebreab et al. (24)] indicate the energy matrix able to mitigate this impact on climate change (i), eutrophication (ii), and acidification (iii) depending on the reference considered to account soybean oil production.

The simulation to estimate the minimum amount of energy matrix necessary to mitigate the environmental cost of producing and transporting the enzyme considered that soybean oil was replaced entirely by corn in the formulas to estimate the environmental cost of using β-mannanase in feeds for pigs and poultry, as the changes in soybean meal depend on the energy matrix used in the formulation (Figure 6). Considering that the inclusion of β-mannanase is 300 g per ton of feed and the functional unit of 1 kg of manufactured feed, the impact associated with the enzyme is 6 g of CO_2_-eq, 0.0151 g of PO_4_-eq, and 0.0262 g of SO_2_-eq (indicated by an A within each panel). When the soybean oil impact is estimated considering Ecoinvent references, the production of soybean oil (1 kg at the feed mill gate) was associated with the emission of 6,008 g of CO_2_-eq, 9.71 g of PO_4_-eq, and 5.00 g of SO_2_-eq. The mitigation of climate change occurred at an energy matrix of 5.4 kcal for pigs and 5.9 kcal for broilers (indicated by a B within each panel), while eutrophication was mitigated at 17 kcal for pigs and 18 kcal for broilers. However, the mitigation may occur even with a lower energy matrix (indicated by C and D within each panel) if other references were used to characterize the environmental impacts of soybean oil. It is worth mentioning that, in real-life conditions, it is recommended to use a percentage of soybean oil to stimulate the feed's palatability and improve mixing conditions.

## Discussion

With regards to the environmental impacts of using β-mannanase in feeds, our results are similar to those obtained by Nielsen et al. (5) when describing the phytase production (1,900 g of CO_2_-eq, 2.20 g of PO_4_-eq, and 4.80 g of SO_2_-eq, 1 kg at the enzyme producer). On the other hand, for the environmental impacts of grain production, crop management practices and expansion rates varied among the Brazilian regions simulated in this study, and so did results. Alvarenga et al. (17) reported equivalent environmental impacts for broiler chicken diets produced in Brazil, but with a slighter lower climate change impact, especially for the CW-CW scenario. Cherubini et al. (25) also reported equivalent impacts in terms of carbon footprint when assessing diets for finishing pigs in Brazil. Both climate change and eutrophication impacts estimated in the current study were also comparable to those obtained by van der Werf et al. (26) and Mosnier et al. (19), who assessed finishing pig diets produced in France using Brazilian soybean. On the other hand, results concerning the use of β-mannanase on acidification were not consistent among feeds once the acidification impact was heavily associated with the feed formula being considered in the simulation. In other words, for some feeds, there was a slight increase in the acidification impact following β-mannanase supplementation; for other feeds, it was quite the opposite. For growing pig diets, the acidification impact was greater when considering the 90 kcal of ME/kg of feed matrix. However, for finishing pig diets, soybean oil was removed from the feed formula for this matrix, lowering its impact on acidification. The acidification impact varied with the corn/soybean proportion. Both climate change and eutrophication impacts are greater for soybean than corn. On the other hand, the acidification impact is greater for corn, especially the one from the Southern region. As this corn/soybean ratio varies from region to region, the same change (%) in the formula ends up increasing or mitigating the overall acidification impact.

For growing-finishing pig feeds, the energy matrix of β-mannanase was chosen based on the most common values applied to the Brazilian industry (45 or 90 kcal of ME/kg of feed). For feeds that do not use exogenous enzymes other than β-mannanase, the saving of 90 kcal of ME/kg of feed provided by β-mannanase is commonly considered. On the other hand, for feeds that include a mix of enzymes, a 45 kcal of ME/kg of feed matrix is more appropriate. Broiler feeds under Brazilian feeding programs commonly include multiple exogenous enzymes when formulated. Therefore, we only considered a 45 kcal of ME/kg of feed matrix in our simulations for broilers. Despite the differences among the several enzyme supplementation strategies available for nutritionists, all simulated values are much higher than the minimum matrix necessary to mitigate the environmental impact associated with the enzyme incorporation in the formula (i.e., 5.4 kcal of ME for pigs and 5.9 kcal of ME for broilers to mitigate the climate change impact, or 17 kcal of ME for pigs and 18 kcal of ME for broilers considering the eutrophication impact). The use of an energy matrix of 45 kcal of ME/kg of feed associated with β-mannanase reduced by 0.90 and 0.84 percent points the inclusion of soybean oil in feed formulas for pigs and broilers, respectively. Considering the recent price conditions in Brazil, the space created in the formula by the reduction of oil use is occupied by corn. However, the average inclusion of corn in the feeds with β-mannanase increased by 0.99 and 0.95 percent points for pigs and broilers, respectively, compared to control diets. Corn variation was higher than the reduction in oil use because soybean meal inclusion was also reduced (on average, a reduction of 0.12 percent points). From an environmental standpoint, these variations are favorable as soybean meal and soybean oil are associated with a higher environmental impact than corn. Formulating feeds for pigs and poultry through liquid energy is a reality in some countries. However, this is not the case in Brazil and therefore it was not included in our analyses.

Overall, the greater environmental impact observed for soybean oil compared to corn is mainly because of the greater amount of resources needed to obtain this ingredient, such as land and fertilizers. The impact of soybean oil considered in this study was based on the Ecoinvent database (v. 3.0, Swiss Center for Life Cycle Inventories, Dübendorf, Switzerland), which is lower than other references for the same product. Simulating the production of 1 kg of feed, the changes in feed formula (average reduction of soybean oil and meal, and increasing corn use) caused by β-mannanase can prevent the emission of 55 g of CO_2_-eq and 0.11 g of PO_4_-eq. If values from AgriFootPrint (v. 5.0, Blonk Consultants, Gouda, The Netherlands) were considered, the mitigation is raised to 112 g of CO_2_-eq and 0.13 g of PO_4_-eq. If the impact estimated by Kebreab et al. (24) for Brazilian soybean oil would be considered, the mitigation associated with β-mannanase could reach 140 g of CO_2_-eq per kg of feed produced. These differences are mostly due to the grain production scenario considered in the database (Southern or Central-West origin).

Differences in the environmental impact of β-mannanase supplementation between pig and poultry feeding programs are mostly due to the ingredients included in diet formulations as each ingredient has it is own environmental impact. However, the pig and poultry sectors share some similarities not only from an organizational point of view but also from an environmental one. Pig and poultry production systems have been pointed out as large contributors to environmental impacts, such as climate change, eutrophication, and acidification (3, 27). Feeding both pigs and poultry requires tremendous amounts of feed resources, especially rich in protein and/or energy, with several studies indicating it as the most important source of environmental impact (2, 4). Novel feeding strategies are thus needed to tackle the challenges of these sectors. Our study has shown that β-mannanase supplementation can be considered as an eco-friendly feed strategy to reduce the environmental impacts of pig and poultry feeding programs. This was mostly because β-mannanase is a nutrient-sparing enzyme that breaks down β-mannans, leading to an increase in energy-use efficiency for both sectors.

## Conclusion

β-mannanase supplementation reduced the amount of soybean oil in feed formulas, which is associated with high environmental impacts. Consequently, the potential impacts of climate change and eutrophication associated with producing feeds for pigs and broilers were substantially mitigated. These results suggest that β-mannanase supplementation is an eco-friendly feed strategy to reduce the environmental impacts of pig and poultry feeding programs. As feeding accounts for most of the environmental impacts associated with pig and poultry production, strategies such as β-mannanase supplementation that mitigate these impacts are desired. This feeding strategy improves the overall sustainability of pig and poultry production systems by increasing energy-use efficiency. It is worth mentioning that the β-mannanase supplementation described in this study is only one way to address the environmental impacts of feeding pigs and broilers. Several other approaches and techniques must be considered in an integrated way toward more sustainable animal systems.

## Data Availability Statement

The raw data supporting the conclusions of this article will be made available by the authors, without undue reservation.

## Author Contributions

FH and IA assisted with data analysis, interpreted results, prepared tables and figures, and drafted the manuscript. MK assisted with data analysis, interpretation and discussion of results. GG, AR, JV, and M-PL-M were involved in the interpretation and discussion of results. All authors have contributed to this research and approved the final version of the manuscript.

## Funding

The authors would like to thank Coordenação de Aperfeiçoamento de Pessoal de Nível Superior (CAPES) and Conselho Nacional de Desenvolvimento Científico e Tecnológico (CNPq) from Brazil for funding this research, and the in-kind support from Elanco Animal Health for sharing the β-mannanase *Hemicell*™ *HT* production inventory.

## Conflict of Interest

The authors declare that this study received in-kind support from Elanco Animal Health, IN, United States, which shared the β-mannanase *Hemicell*^TM^ *HT* production inventory. In addition, MK and JV are employed by the company Elanco Animal Health. The remaining authors declare that the research was conducted in the absence of any commercial or financial relationships that could be perceived as a potential conflict of interest.

## Publisher's Note

All claims expressed in this article are solely those of the authors and do not necessarily represent those of their affiliated organizations, or those of the publisher, the editors and the reviewers. Any product that may be evaluated in this article, or claim that may be made by its manufacturer, is not guaranteed or endorsed by the publisher.

## Abbreviations

CO_2_-eq: carbon dioxide equivalent
CW: Central-West region
CW-CW: the scenario in which only grains from the Central-West region were used to produce the feed
CW-SO: the scenario in which soybeans from the Central-West region and corn from the Southern region were used to produce the feed
DE: digestible energy
LCA: life-cycle assessment
ME: metabolizable energy
SO_2_-eq: sulfur dioxide equivalent
SO: Southern region
SO-SO: the scenario in which only grains from the Southern region were used to produce the feed
PO_4_-eq: phosphate equivalent.
